# What motivates antibiotic dispensing in accredited drug dispensing outlets in Tanzania? A qualitative study

**DOI:** 10.1186/s13756-015-0073-4

**Published:** 2015-07-21

**Authors:** Angel Dillip, Martha Embrey, Elizabeth Shekalaghe, Dennis Ross-Degnan, Catherine Vialle-Valentin, Suleiman Kimatta, Jafary Liana, Edmund Rutta, Richard Valimba, John Chalker

**Affiliations:** Ifakara Health Institute, P.O. Box 78373, Dar es Salaam, Tanzania; Management Sciences for Health, Arlington, VA USA; Pharmacy Council, Dar es Salaam, Tanzania; Harvard Medical School and Harvard Pilgrim Health Care Institute, Boston, USA; Management Sciences for Health, Dar es Salaam, Tanzania

**Keywords:** ADDO, Antibiotics, Antimicrobial resistance

## Abstract

**Background:**

Tanzania introduced the accredited drug dispensing outlet (ADDO) program more than a decade ago. Previous evaluations have generally shown that ADDOs meet defined standards of practice better than non-accredited outlets. However, ADDOs still face challenges with overuse of antibiotics for acute respiratory infections (ARI) and simple diarrhea, which contributes to the emergence of drug resistance. This study aimed to explore the attitudes of ADDO owners and dispensers toward antibiotic dispensing and to learn how accreditation has influenced their dispensing behavior.

**Methods:**

The study used a qualitative approach. We conducted in-depth interviews with ADDO owners and dispensers in Ruvuma and Tanga regions where the government implemented the ADDO program under centralized and decentralized approaches, respectively; a secondary aim was to compare differences between the two regions.

**Results:**

Findings indicate that the ADDO program has brought about positive changes in knowledge of dispensing practices. Respondents were able to correctly explain treatment guidelines for ARI and diarrhea. Almost all dispensers and owners indicated that unnecessary use of antibiotics contributed to antimicrobial resistance. Despite this knowledge, translating it to appropriate dispensing practice is still low. Dispensers’ behavior is driven by customer demand, habit (“*mazoea”*), following inappropriate health facility prescriptions, and the need to make a profit. Although the majority of dispensers reported that they had intervened in situations where customers asked for antibiotics unnecessarily, they tended to give in to clients’ requests. Small variations were noted between the two study regions; for example, some dispensers in Ruvuma reported sending clients with incorrect prescriptions back to the health facility, a practice that may reflect regional differences in ADDO implementation and in Integrated Management of Childhood Illness training. Dispensers in rural settings reported more challenges in managing ARI and diarrhea than their urban counterparts did.

**Conclusion:**

To reduce inappropriate antibiotic use, integrated interventions must include communities, health facilities, and ADDOs. Periodic refresher training with an emphasis on communication skills is crucial in helping dispensers deal with customers who demand antibiotics. Responsible authorities should ensure that ADDOs always have the necessary tools and resources available.

## Background

Tanzania implemented the accredited drug dispensing outlet (ADDO) program in 2003 [1–3]. The program aims to improve access to affordable, quality medicines and pharmaceutical services, especially in rural and underserved areas with few or no registered pharmacies [1, 4]. The ADDO program was created as a public-private partnership responding to an assessment that showed important gaps in availability and quality of essential medicine in the country [4]. The program relies on a combination of owner and dispenser training, regulatory enforcement, government accreditation, and business incentives, such as being allowed to sell a wider selection of medicines than other shops, including select antibiotics. To become accredited, shop premises must meet infrastructure standards, and dispensers must pass a training program and an examination, among other requirements. The government of Tanzania has now rolled out the ADDO program in all regions of Tanzania’s mainland with more than 9000 outlets accredited or awaiting accreditation and 19,000 dispensers trained.

As part of their accreditation process, ADDO dispensers receive training on Integrated Management of Childhood Illness (IMCI), which includes principles of effective ARI and diarrhea management in children. Antibiotics are indicated for pneumonia and severe pneumonia, but not for acute respiratory tract infections (ARIs) [5, 6]. The guidelines also recommend the use of oral rehydration solution (ORS) and zinc supplements for watery diarrhea, rather than antibiotics, which are only used to manage chronic or bloody diarrhea [5, 6].

Unnecessary use of antibiotics is ineffective and costly and contributes to antimicrobial resistance (AMR) [7]. The global impact of AMR remains unknown, however, the challenges are reportedly greater in low- and middle-income countries, where antibiotics are often purchased without prescription and prescribed and dispensed inappropriately [8–12]. The burden of AMR in African countries has been difficult to estimate due to lack of surveillance systems and reliable data, but a review of the literature indicates that Tanzania is seeing a rise in multi-drug resistant organisms, particularly *Escherichia coli, Klebsiella pneumoniae, Staphylococcus aureus, Vibrio cholera*, and non-typhoid *Salmonella* [13, 14].

Since the beginning of the ADDO program in Tanzania, several evaluations have generally shown them to perform better than non-accredited outlets, according to defined standards of practice [15–18]. However, overuse of antibiotics for ARIs and simple diarrhea in the community remains problematic [8, 9]. In a recent study using mystery shoppers presenting as parents of a two-year-old child, ADDO dispensers sold antibiotics to 34 % of shoppers describing the child’s mild ARI signs and symptoms, while dispensers sold an antibiotic to 85 % of shoppers presenting the same symptoms and a specific request for co-trimoxazole [8].

This study aimed to explore ADDO dispensers’ and owners’ perceptions, knowledge, and attitudes regarding medicine use, AMR, treatment guidelines, and antibiotic dispensing in way that characterizes the motivations that underlie unnecessary antibiotic use.

## Methods

This was a qualitative study using in-depth interviews. The study was rooted in principles of grounded theory [19]. We interpreted the data as we collected it in the field and used results to revise the interview guides on an ongoing basis.

### Setting

In the study, we compared two regions, Tanga and Ruvuma, where ADDO implementation was done using two different models. In Tanga, program implementation was carried out as part of the national ADDO rollout (2009–2010) using a decentralized approach with local trainers and implementers, while in the Ruvuma region (2003–2004), which was the location of the program pilot, Management Sciences for Health and the Tanzania Food and Drugs Authority directly set up the program under a centralized model. Centralization and decentralization approaches are further explained by Rutta and colleagues [20]. Our secondary aim was to explore differences between the two regions in antibiotic dispensing and use and in knowledge about AMR and treatment guidelines. In each region, we purposively selected two districts to explore potential rural/urban differences: Muheza (rural) and Tanga urban in the Tanga region and Mbinga (rural) and Songea urban in the Ruvuma region.

### Research team and reflexivity

Prior to data collection, two experienced Tanzanian research assistants were recruited and trained to conduct pilot interviews in Dar es Salaam. Together with the team leader, who is a qualitative research expert, each research assistant conducted two interviews with ADDO dispensers. Based on this experience, we revised the in-depth interview guide to use for the actual data collection in the two study regions.

The research assistants carried out the field work in Swahili; their roles included recruiting participants, seeking informed consent, conducting data collection, and writing field notes. The interviewers elicited accounts of antibiotic dispensing practices and encouraged discussion around issues related to knowledge of treatment guidelines and motivations behind antibiotic dispensing, medicine use, and AMR. Study participants did not know the interviewers, who were introduced on the day of the data collection by a district pharmacist.

### Participant selection

The study included three types of participants: ADDO owners who are also dispensers (“owner-dispenser”), ADDO owners who are not dispensers (“owner-not dispenser”), and ADDO dispensers who are not owners (“dispenser-not owner”). The study participants were purposively sampled to ensure that we obtained relevant information related to the study objectives and to highlight possible differences in responses among the three groups. We determined our sample size using saturation sampling [21, 22]. We planned to conduct at least seven interviews with each type of participant in each study district.

### Data collection

Over a three-week period, we conducted face-to-face interviews using a semi-structured interview guide. We interviewed two groups of respondents (owner-dispensers and dispenser-not owners) in their outlets. The process was flexible enough to allow customers to access services in the ADDO during the interviews process. With the assistance of district pharmacists, we used phone calls and other means to trace ADDO owner-not dispensers, who were not available in outlets at the time of data collection. Interviews were recorded, and the research assistants also took notes during interviews to expand later. Collected data were systematically analyzed in sequence and used to inform subsequent data collection.

## Ethical approval

The study received ethical approval from the National Institute of Medical Research in Tanzania. Moreover, we obtained oral and written consent at the start of each interview.

## Analysis and reporting

Data were managed using NVIVO software [23]. We coded and checked interviews for coding consistency using a framework of relevant themes that addressed key issues defined by our study objectives. The three main themes covered topics related to knowledge of treatment guidelines, motivation behind antibiotic dispensing, and medicine use and AMR.

After analysis, we combined findings from two groups of participants who dispense, namely ADDO owner-dispensers and ADDO dispenser-not owners, because the findings for these groups were similar; we refer to both collectively as ADDO dispensers. In the results, we note differences between these groups.

## Results

Our study sample included 84 respondents, 51 (61 %) of whom were women and 33 (39 %) men. As planned, we conducted seven interviews in each study group and in each study district, except in Mbinga, where we interviewed six ADDO owner-dispensers and eight ADDO dispenser-not owners because of challenges in tracking down ADDO owners who are also dispensers.

Results are presented in the following five categories: 1) knowledge about treatment guidelines, 2) motivation behind antibiotic dispensing, 3) medicine use and antimicrobial resistance, 4) referrals for pneumonia cases, and 5) for the ADDO owners, perceptions about AMR and their role in the community.

### Knowledge about treatment guidelines

We assessed knowledge of disease management in ADDOs as per the IMCI and adult training manual for ADDO dispensers [24]. Dispensers were asked to explain treatment guidelines for ARI and diarrheal diseases.

#### Severe pneumonia

For treatment of severe pneumonia, the standard treatment protocol for ADDO dispensers recommends providing an antibiotic and then immediately referring the case to a health facility.

Nearly all ADDO dispensers (55/56) in all study regions were able to correctly explain the management of severe pneumonia, “*I don’t handle severe pneumonia here. Health facility is very close, when I receive such cases, I immediately refer a patient to health facility, although I currently don’t have referral forms”* (dispenser and owner, Tanga). Another respondent said, “*I will give her antibiotics and insist that she finishes the dose. I will also advise her to go to health facility if no improvements”* (dispenser and owner, Mbinga district).

We observed several differences in knowledge between rural and urban settings. More owner-dispensers in the urban areas of Tanga and Songea (10/14) correctly explained the management of severe pneumonia than those in the rural Mbinga and Muheza districts (7/13).

#### Pneumonia

Standard treatment protocols for pneumonia at the ADDO require provision of antibiotics and referral to a health facility in the case of danger signs, such as fast or difficult breathing. As with severe pneumonia, a higher proportion of dispensers (27/28) in the urban areas of Tanga and Songea knew how to manage pneumonia compared to those working in the rural areas of Mbinga and Muheza (21/27). “*If it is a child, I will give her antibiotic syrup, and if it is an adult, I will provide her with antibiotics in the form of tablets”* (dispenser and owner, Songea urban).

#### Non-pneumonia cold/cough

The standard treatment for non-pneumonia cough at the ADDO is paracetamol for fever and headache and an appropriate remedy to soothe the cough. We observed discrepancies between districts. While the majority of ADDO dispensers appropriately explained treatment of non-pneumonia cold/coughs (21/28), four of seven dispensers in Muheza district reported that they would dispense antibiotics to clients with non-pneumonia coughs. “*I will give her Septrin or amoxicillin or Pen-V”* (dispenser, Muheza district).

#### Non-bloody diarrhea

The standard treatment for non-bloody diarrhea at ADDO is ORS and zinc—not antibiotics. Interestingly, all dispensers in the Tanga urban and Mbinga rural districts understood how to correctly manage non-bloody diarrhea, while fewer in Songea urban (4/7) and Muheza rural (4/7) knew the recommended practice. “*Because with diarrhea, one loses a lot of water, so I will provide him with ORS; you cannot provide antibiotics, it will not treat the patient”* (dispenser, Mbinga district). All owner-dispensers in all study districts knew the correct management practices with the exception of Muheza (4/7).

#### Bloody diarrhea

Blood in the stool likely indicates dysentery for which standard treatment protocols recommend antibiotics. Knowledge of the recommended treatment was also quite high; almost all respondents (54/56) correctly explained how ADDO dispensers should manage bloody diarrhea. “*For someone who is having bloody diarrhea, he definitely has bacteria that goes and destroy intestines; I would dispense an antibiotic like erythromycin that will treat the patient”* (dispenser, Muheza)*.*

### Motivation behind unnecessary antibiotic dispensing

Given the generally widespread knowledge about correct practices for these illnesses, we sought to learn from dispensers why they often dispense antibiotics for non-pneumonia coughs and simple diarrhea. While acknowledging continued overuse, almost all respondents felt that unnecessary antibiotic dispensing had significantly declined compared to the time before drug shops were accredited. We noted small variations in responses across regions, particularly regarding the specific reasons for unnecessary antibiotic dispensing.

#### Customer preference

86 % of dispensers interviewed (48/56) reported that customers prefer to purchase antibiotics for non-pneumonia coughs and for non-bloody diarrhea (41/56, 73 %). “*You know the clients communicate among themselves; they tell each other that antibiotics treat cough, so when they come here, they specifically ask for antibiotics*” (dispenser, Tanga urban)*.* Half of ADDO dispensers claimed to intervene by educating clients on the correct treatment for non-pneumonia coughs/colds and simple diarrhea and on the fact that antibiotics do not treat such health problems. However, respondents also admitted that when clients demand what they want that dispensers frequently end up adhering to their wishes.

#### Dispense according to health facility prescription

Another frequently mentioned reason for dispensing antibiotics for non-pneumonia coughs was following the prescriptions they get from health facilities. This was recounted by 23 of 29 (79 %) dispensers and 12 of 27 (44 %) owner- dispensers for non-pneumonia cough and 18 of 29 (62 %) dispensers and 7 of 27 (26 %) owner- dispensers for non-bloody diarrhea.

We wanted to learn if dispensers ever intervene when they receive an incorrect health facility prescription. Less than half of dispensers reported sending the clients back to the facility for a new prescription or asking them to buy the prescribed medicines from other ADDOs. More dispensers in Ruvuma region than Tanga region said they challenged wrong prescriptions from health facilities, but not always with positive results. *“Yes, we send them back to health facility, but again they insult us that we are just dispensers, we don’t have any medical knowledge and that what the doctor has decided we should follow; it is not easy”* (dispenser and owner, Songea urban). *“It is difficult to challenge what the doctor has prescribed; people respect doctors a lot. A day before yesterday, a child aged three months was brought with antibiotic prescribed for cough problem. I refused to provide an antibiotic. I sent the parent back to the health facility. I am not sure if she went back or not”* (dispenser and owner, Songea urban). Sixty-eight percent of respondents (38/56) reported that they always adhere to doctors’ prescriptions. *“You cannot go against the doctor’s prescription. He has the last say, even when we see the prescription is wrong; you just give the client what is written”* (dispenser, Muheza district).

#### Business reasons

Respondents also reported that they sometimes dispense antibiotics inappropriately to finish the shop’s medicine stock or to make more profit on expensive medicines (i.e., antibiotics). Seventy-seven percent of dispensers (43/56) reported these reasons for dispensing antibiotics for non-pneumonia coughs and 71 % (40/56) for non-bloody diarrhea. Of note, many dispensers accused ADDO owner-dispensers of these practices to increase income. Some dispenser-not owners (5/29) admitted to receiving pressure from their owners to sell more antibiotics—although only 9 of 27 owner-dispensers reported ever having had heard of the practice.

#### Common practice

Over three-quarters of dispenser-not owners (23/29) admitted that it is just common practice (“*mazoeza”*) for them to dispense antibiotics for non-pneumonia coughs. A smaller proportion of owner-dispensers admitted this reason (11/27).

### Medicine use and antimicrobial resistance

More than 90 % of study respondents reported that they saw AMR as a consequence of unnecessary use of antibiotics. Some expressed the view that when antibiotics are administered unnecessarily, the medicine can lose its strength and fail to treat. “*It causes drug resistance and kills white blood cells”* (dispenser and owner, Mbinga district). “*You can be surprised, someone will use and use and use, but he will not get treated, because the medicine is already resistant”* (dispenser, Tanga urban).

### Failure to refer pneumonia cases

We questioned dispensers about their reasons for not providing referrals for pneumonia cases, as recommended in their training. We noted variations in responses between the two regions. More Ruvuma dispensers (23/28) than their Tanga counterparts (12/27) disclosed that business is the main factor driving their referral decisions, because referral means no income. “*They want to make money, raise income….that is the reason referrals are not provided”* (dispenser, Songea urban). Other reasons reported included laziness and community affordability, as the following comments illustrate: *“There are times when dispensers feel lazy to fill referral forms so they just dispense medicines*” (dispenser and owner, Songea urban). *“Other customers may come and want to take medicine on loan basis, so if you tell them to go to health facility, they will not go because they do not have money, so you just give them medicine”* (dispenser and owner, Songea urban).

In Tanga region, respondents mentioned that there are no referral forms at the outlets, while others felt that they did not need to refer pneumonia patients because they have satisfactory knowledge about how to treat the condition. Fear of losing trust from the community was another factor reported in rural Muheza and Mbinga. “*People trust us as professionals, so when you give referral, they question your ability, and your status might go down…besides we have all medicines to treat severe pneumonia”* (owner and dispenser, Muheza).

### ADDO owners’ perceived roles

We were specifically interested to learn from ADDO owner-not dispensers about their perceived roles in the community and their perceptions about antibiotic dispensing and AMR.

Of 28 owners interviewed, 26 (93 %) perceived their outlets as a place where community members can access medical services and advice. They felt that their main responsibilities as owners were to ensure that essential medicines are available in their outlets, that medicines are of good quality, that expired medicines are not on the shelves, and that their business increases.

When asked what kinds of medicines they sold most in their outlets, antibiotics were mentioned by more than half of owners, followed by antimalarials and painkillers. Sixty-one percent of owners (17/28) attributed the practice of dispensing antibiotics without doctor’s advice to customer preference. “*You know, we have problems with customers. When they come, they always demand antibiotics”* (owner, Tanga urban).

Almost all owners mentioned antimicrobial resistance as one effect of the unnecessary use of antibiotics. Moreover, 95 % of owners (26/28) reported that their dispensers manage various health problems by following treatment guidelines.

Regarding changes in antibiotic sales after the ADDO program was implemented, respondents reported mixed views. Overall, 17 of 28 (61 %) perceived that antibiotic sales increased after the ADDO program began because antibiotics were previously not allowed to be sold in the shops. “*It is true after ADDO program, we now have more antibiotics which we are allowed to sell compared to the period during Part II drug shops; it is not that they are sold unnecessarily, but because they are there, people can have easy access”* (owner, Songea urban).

The remaining owners (11/28, 39 %) thought that antibiotic sales are lower now because they need to be dispensed with doctor’s prescriptions. “*You know before the ADDO program, dispensers were selling antibiotics illegally, but now, it is hard to be sold an antibiotic without prescription”* (owner, Mbinga).

## Discussion

Our findings suggest that the ADDO program has brought about constructive changes in antibiotic knowledge and dispensing practices. The majority of dispensers understand treatment guidelines for ARI and diarrheal diseases, with the exception of a small number of dispensers in rural areas.

Respondents indicated that inappropriate antibiotic dispensing behavior is driven primarily by customer demand, common practice, following health facility prescriptions, and profit. However, ADDO accreditation appears to have introduced some countervailing influence; for example, dispensers report that they sometimes intervene in situations where customers request unnecessary antibiotics. However, the positive influence of ADDO training and accreditation alone has not outweighed the factors that drive inappropriate antibiotic dispensing, including patient demand and irrational prescribing in facilities. Programs to reduce unnecessary antibiotic use will therefore need to target the community/clients and public health facility prescribers as well as ADDO dispensers and owners.

We did not note major differences between the two study regions, Tanga and Ruvuma, with the exception that more dispensers in Ruvuma reported a willingness to send clients with incorrect prescriptions back to the health facility. One reason for this might be that Ruvuma was the pilot region for the program, and therefore received concerted attention, while ADDO implementation in Tanga was done through a decentralized approach with less-intensive training. An earlier study to assess stakeholders’ satisfaction with centralized versus decentralized ADDO implementation approaches revealed that some thought the quality of the decentralized training during the national rollout was lower [25]. Stakeholders felt that the training duration in the decentralized model was too short to cover all topics, resulting in a lower level of understanding among trainees [25]. Nevertheless, the majority of dispensers appear to adhere to facility prescriptions even when they run counter to the training they received on managing non-pneumonia coughs and simple diarrhea. Antibiotic dispensing practices in the two regions may also differ because ADDO dispensers in the Ruvuma region received more-intensive IMCI training that included rational medicines use for the key common childhood conditions: malaria, ARI, and diarrhea [26].

We observed some differences in responses between ADDO dispensers from rural and urban settings; for example, rural dispensers noted fear of losing the trust of their communities and their desire to maintain their status as professionals. Because rural areas have fewer medicine outlets and health facilities, access to medical services is more limited. Once clients are used to visiting a specific outlet in their village, they may develop stronger relationships with the dispensers. Dispensers see themselves as health professionals, and they strive to maintain community respect, which might encourage them to try to treat some health problems that are beyond their ability. Moreover, distance to health facilities is always more of a challenge in rural areas, which influences patients’ choice to visit nearby outlets.

The fact that few owner-dispensers reported filling incorrect prescriptions from health facilities may be due to their professional background. Most owner-dispensers in Tanzanian villages are retired clinicians, so they may be unwilling to comment openly on this issue at the risk of sounding disloyal to their profession.

## Strengths and limitations

This research had several strengths. The study approach was culturally anchored, with data collection undertaken in Swahili by local research assistants who were accustomed to the local environment and were familiar with the study area. The same researchers took part in translation and analyses. Moreover, all respondents agreed to take part in the study and were willing to speak freely about potentially sensitive issues. Based on responses from participants, we strongly feel that we reached data saturation.

Nevertheless, the research also had limitations. We experienced distance-related challenges in some study districts, and we ended up not accessing all participants in each study group as planned. We also carried out the study in four purposively selected districts, so the findings may not be representative of the entire Tanzanian setting. Like any in-depth interviews, our study may be subject to social desirability bias, in that respondents might have told us what they expected we wanted to hear, especially on issues related to treatment knowledge; however, using well-trained social research scientists with good probing skills likely minimized the extent of the bias.

## Recommendations

Based on the issues related to dispensing practices raised by respondents in this study, we recommend several actions to reduce clinically inappropriate antibiotic use in ADDOs.

Given that customer preference and prescribing practices at health facilities appear to be major drivers for inappropriate antibiotic dispensing in ADDOs, the Pharmacy Council, which has regulatory authority over ADDOs, should help coordinate interventions targeting health facility providers, community members, and ADDO staff. Health facility interventions should aim to ensure proper guideline-based antibiotic prescribing for common illnesses. Community social marketing interventions should emphasize the waste of household resources when buying unneeded antibiotics, the harmful long-term effects of unnecessary use, the importance of taking a full course of therapy, and the basic services customers should expect to receive at ADDOs and health facilities for ARIs and diarrhea. Flyers with important educational messages such as *Septrin does not cure coughs* should be designed and displayed at ADDOs and health facilities to support changes in staff practices; related messages should also be communicated via radio and television.

Refresher training for dispensers is important. Periodic training should focus on reminding dispensers of the harmful effects of unnecessary antibiotic use. The training should emphasize that people trust ADDO services, and that as dispensers, they have a unique role in helping reduce morbidity and mortality from childhood respiratory illness and diarrhea as well as in ensuring the sustainability of the ADDO program. Training on communication skills may help dispensers negotiate conflict more effectively with clients who demand antibiotics inappropriately. In addition, authorities need to ensure that ADDOs have the necessary resources available to support their operations, such as referral forms and dispensing registers.

Antibiotic dispensing is sometimes motivated by financial concerns. Conducting regular mystery shopping studies at ADDOs and sharing the results with dispensers through their associations may encourage appropriate practices and remind dispensers that their practices are being observed. Similarly, frequent monitoring visits by district pharmacists may also be helpful. To reduce the desire to sell more antibiotics to generate sufficient profit to stay in business, ADDO owners should be encouraged to sell a wider range of products than medicines; for example, accredited drug shops in Uganda have successfully added fortified foods to their inventory.

## Conclusion

Study findings indicate that the ADDO program has had a positive influence on dispensers’ knowledge of treatment guidelines for acute respiratory infection and diarrhea. The majority of dispensers and owners interviewed knew the effects of unnecessary use of antibiotics. However, the dispensers’ translation of correct knowledge into appropriate attitudes and dispensing practice was sometimes driven by customer demand, habits, following inappropriate health facility prescriptions, and profit. Future interventions to address irrational antibiotic use should target consumers, ADDO dispensers, and staff at heath facilities.
